# Effect of hydrofluoric acid concentration and aging on the bond strength ceramics to a resin cement

**DOI:** 10.1590/0103-6440202405669

**Published:** 2024-07-22

**Authors:** Bruno Delgado Clerot, Lourenço Correr-Sobrinho, Milena Bandini, Evaldo Pinheiro, Fernanda Midori Tsuzuki, Rafael Rocha Pacheco, Ana Rosa Costa

**Affiliations:** 1 Department of Orthodontics, Graduate Program in Orthodontics - UNIARARAS, Universidade de Araras, SP, Brazil; 2 Department of Restorative Dentistry, Dental Materials Division, Piracicaba Dental School, UNICAMP, State University of Campinas, Piracicaba, SP, Brazil; 3 Department of Restorative Sciences, The Dental College of Georgia at Augusta University, Augusta, GA, USA

**Keywords:** Cytotoxicity, Cell viability, Raman Spectroscopy, Human Dental Pulp Stem Cells, self-etching adhesive bonding agents

## Abstract

This study evaluated the influence of hydrofluoric acid (HF) concentration and thermal cycling on the microshear bond strength (µSBS) of a resin luting agent to IPS e.max® CAD and Rosetta® SM. Ceramic specimens (12.0 x 14.0 x 1.5mm) were randomized into 8 groups (n=10) according to HF concentration, commercial brand, and aging. Immediately after polishing, and etching, all specimens were silanized and a layer of adhesive was applied. A PVS mold of 3 mm thickness and 10mm diameter with (four) 1.0mm holes was fabricated, placed on each specimen, and then filled with a resin luting agent. Half of the specimens were subjected to the µSBS test using an Instron at a speed of 1.0 mm/min, following a 24-hour storage in deionized water at 37ºC. The remaining specimens were subjected to thermal cycling (5ºC-55ºC, 30 seconds per bath) and µSBS. The data were evaluated utilizing a three-way ANOVA and Tukey's post-hoc test (α=0.05). Significant differences were found for HF concentration and aging (p<0.0001). No significant difference in µSBS was found for commercial brands (p=0.085). The interaction between brand and HF concentration (p=0.358), brand and aging (p=0.135), and HF concentration and aging (p=0.138) were not statistically significant. The triple interaction among these factors was not statistically significant (p=0.610). In conclusion, the bond strength is affected by the HF concentration. No statistical difference was observed between the two ceramics. Thermal cycling significantly reduced µSBS.

## Resumo

Este estudo avaliou a influência da concentração do ácido fluorídrico (AF) e da ciclagem térmica na resistência de união ao microcisalhamento (RUµC) de um cimento resinoso para IPS e.max® CAD e Rosetta® SM. Espécimes cerâmicos (12,0 x 14,0 x 1,5mm) foram divididos em 8 grupos (n=10) de acordo com concentração do HF, marca comercial e envelhecimento. Imediatamente após o polimento e condicionamento ácido, todos os espécimes foram silanizados e uma camada de adesivo foi aplicada. Um molde PVS de 3 mm de espessura e 10 mm de diâmetro com (quatro) orifícios de 1,0 mm foi confeccionado, colocado em cada espécime e preenchido com o cimento resinoso. Metade dos espécimes foi submetida ao teste RUµC na Instron a velocidade de 1,0 mm/min, após 24 horas de armazenamento em água deionizada a 37ºC. Os espécimes restantes foram submetidos a ciclagem térmica (5ºC-55ºC, 30 segundos por banho) e a RUµC. Os dados foram avaliados por ANOVA de três fatores e ao teste post-hoc de Tukey (α=0,05). Diferenças significativas foram encontradas para concentração de HF e envelhecimento (p<0,0001). Nenhuma diferença significativa na RUµC foi encontrada para cada marca comercial (p=0,085). A interação entre marca comercial e a concentração do HF (p=0,358), marca comercial e envelhecimento (p=0,135) e concentração do HF e envelhecimento (p=0,138) não foram estatisticamente significativas. A tripla interação entre esses fatores não foi estatisticamente significativa (p=0,610). Concluindo, a resistência de união é afetada pela concentração de HF. Não foi observada diferença estatística entre as duas cerâmicas. A ciclagem térmica reduziu significativamente a resistência de união ao microcisalhamento.

## Introduction

As the demand for esthetic restorative procedures has increased over the past decade, numerous dental techniques and materials have been developed. Glass ceramics have been used to restore teeth with carious lesions or fractures, as well as to replace teeth with agenesis or clinically poor restorations. These materials have shown biocompatibility, color stability, excellent mechanical properties, chemical stability, radiopacity, low thermal conductivity, and outstanding tissue mimicry ^1^ ^,^ ^2^. Among the numerous ceramics used in restorative dentistry, lithium disilicate-based glass-ceramics have become the preferred material due to their superior mechanical properties, esthetic results, and satisfactory bond strength to resin luting agents when subjected to the proper surface treatment ^3^ ^,^ ^4^. Additionally, the ability to mill a ceramic restoration immediately after scanning a prepared tooth or die stone model using CAD-CAM (Computer Assisted Design / Computer Assisted Manufacturing) technology is a significant advantage that contributes to clinical acceptance. A single crown can be milled and cemented onto a tooth within an hour ^5^ ^,^ ^6^.

In 1998, the first generation of glass ceramic reinforced with lithium disilicate (IPS Empress® 2 by Ivoclar Vivadent, Schaan, Liechtenstein) was introduced to the market. The second generation of pressed lithium disilicate reinforced glass ceramic (IPS e.max® Press by Ivoclar Vivadent) was introduced in 2006, with enhanced mechanical and optical properties ^7^. Eventually, after the IPS e.max® Press patent expired, other companies developed ceramic materials with comparable properties, such as Rosetta® SM (Rosetta, Hass, Gangneung, Korea), GC Initial™ (GC Corporation, Tokyo, Japan), Aidite® (Shenzhen, Guangdong, China), IRIS (Tianjin, Mainland, China), and T-lithium (Talmax, Curitiba, Brazil) ^8^. According to the manufacturers, the mechanical, morphological, and structural properties of these ceramics are comparable to those of the IPS e.max® Press system ^4^.

Glass-ceramics can be bonded to tooth substrates; however, the longevity of the bond depends on the surface treatment employed. Commonly, hydrofluoric acid (HF) is used to modify the surface by dissolving the glassy phase of the ceramic ^4^ ^,^ ^9^ ^,^ ^10^ ^,^ ^11^ ^,^ ^12^. Consequently, an increased surface area with micromechanical retentions serves as a localized site for the mechanical interlocking of the resin luting agent. In addition, an increase in surface energy improves wetting ^2^ ^,^ ^9^ ^,^ ^11^ ^,^ ^13^ ^,^ ^14^ ^,^ ^15^ ^16^. The effectiveness of HF can be affected by its concentration, application time, temperature, and dilution ^2^ ^,^ ^9^ ^,^ ^10^ ^,^ ^11^. The manufacturer of IPS e.max® Press recommends etching it for 20 seconds using a 4.8% HF solution. In contrast, numerous in vitro studies have recommended various HF concentrations and etching times, such as 10% for 15 seconds ,10,11, 10% for 20 seconds ^1^ ^,^ ^3^, 9.5% for 60 seconds ^17^, 4.8% for 60 seconds ^18^, 5% for 60 seconds ^19^, and 4.8-5% for 20 seconds ^20^. Moreover, it is recommended to use a coupling agent (silane) immediately after etching, rinsing, and drying, and before applying an adhesive layer. This procedure can improve the bond strength and quality at the ceramic-resin luting agent interface. The silane may also assist the resin luting agent to penetrate deeper into the etched ceramic's irregular surface ^2^.

For restorative procedures using dental ceramics, the quality and durability of the adhesive interface between the ceramic and the resin luting agent are essential ^9^. An optimal interface prevents leakage and can dissipate the stresses from the ceramic restoration to the structure, preventing failures ^21^. When ceramics are exposed to the oral environment in a clinical setting, failures may occur if the bonded interface lacks optimal bond strength and quality. These failures may be caused by thermal, physical, and chemical factors. Thermal cycling is an alternative method for inducing stress and activating factors that can cause bond degradation before mechanical testing ^22^ ^,^ ^23^. Different temperatures and coefficients of expansion between materials can increase interfacial tension and result in adhesive failure ^22^. For better guidance in clinical procedures, a protocol with well-established criteria for bonding varied lithium disilicate-reinforced glass ceramics with an optimal HF concentration is needed. Such a protocol could provide essential information to improve the performance and clinical longevity of restorative dental procedures using these ceramics.

The purpose of this study was to investigate the microshear bond strength (µSBS) of a resin luting agent to two ^2^ lithium disilicate-reinforced glass ceramics after surface treatment with varying HF concentrations and the effects of thermal cycling on the bond strength. The tested research hypotheses were as follows: [1] different HF concentrations will affect µSBS; [2] different ceramics will have similar µSBS values; and [3] thermal cycling will have a negative effect on µSBS for both ceramics.

## Material and Methods

### Specimen fabrication

**Figure 1:**
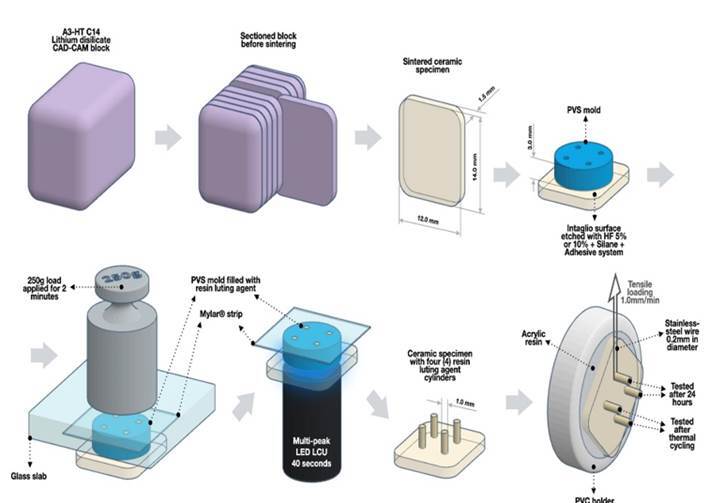
Diagram of µSBS testing methodology

Forty-two 42 ceramic specimens measuring 12.0 mm in width, 14.0 mm in length, and 1.5 mm in thickness (Figure 1) were obtained from each of two ^2^ commercial brands of lithium disilicate reinforced ceramic for CAD-CAM: [EM] IPS e.max® CAD (Ivocar Vivadent, Schaan, Liechtenstein, HT-A3 C14) and [RS] Rosetta® SM (Hass, Gangneung, Korea, HT-A3 C14). The blocks were sectioned (12.0 mm in width, 14.0 mm in length, and 1.7 mm in thickness) using a precision cutting machine (Isomet 1000, Buehler, Lake Bluff, IL, USA) that included a diamond disc (EXTEC; Enfield, CT, USA) at low speed and constant water cooling. Using silicon carbide sandpapers of progressively finer grit sizes (#600, #1200, and #2000, Norton SA, São Paulo, SP, Brazil) under constant water cooling one side of the ceramic surfaces was finished and polished to obtain flat surfaces. The specimens were subjected to a 10-minute ultrasonic bath in water to remove any debris before being randomly assigned to eight ^8^ groups (n=10) based on HF concentration (5 or 10%), commercial brand, and *aging* (Table 1)*.* The specimens were etched using HF (Fórmula & Ação, São Paulo, SP, Brazil) at concentrations of 5% or 10% (based on their assigned group) for 20 seconds, air/water rinse for 1 minute, immersed in an ultrasonic water bath for 1 minute (MaxiClean 750, Unique, Indaiatuba, SP, Brazil), and air-dried for 30 seconds. Using a microbrush, RelyX™ Ceramic Primer (3M ESPE, St. Paul, Minnesota, United States) was actively applied to each ceramic surface for 15 seconds. The solvent was evaporated by applying 60°C (±5°C) hot air from 15 cm away for 45 seconds. After silane application and solvent evaporation, a thin layer of Single Bond Universal (3M ESPE, Sumaré, SP, Brazil) was applied for 20 seconds, followed by gentle air-drying for 10 seconds, and then light-activated using a multi-peak LED light-curing unit (Bluephase® G2, Ivoclar Vivadent, Schaan, Liechtenstein) for 10 seconds with an emitted irradiance of 1,100 mW/cm^2^.

**Table 1:** Distribution of experimental groups according to commercial brand, HF concentration, and aging (24 hours or thermal cycling).

Ceramic	HF concentration	Aging	Groups
IPS e.max® CAD (Ivocar Vivadent, Schaan, Liechtenstein, HT-A3 C14)	5%	24 hours	EM5-
		Thermal cycling	EM5+
	10%	24 hours	EM10-
		Thermal cycling	EM10+
Rosetta® SM (Hass, Gangneung, Korea, HT-A3 C14)	5%	24 hours	RS5-
		Thermal cycling	RS5+
	10%	24 hours	RS10-
		Thermal cycling	RS10+

### Microshear bond strength (µSBS)

Using PVS (Express™ STD, 3M ESPE), 3.0 mm thick and 10.0 mm in diameter molds with four ^4^ holes of 1.0 mm in diameter were made. The molds were positioned on the ceramic specimens and filled with a resin-luting agent (RelyX™ Ultimate, 3M ESPE, shade A2). Then, a Mylar® strip and a glass slab were placed on the mold (filled with resin luting agent) and a 250-gram load was applied for 2 minutes. Following the removal of the load and the glass plate, the resin luting agent was photoactivated through the ceramic for 40 seconds using the multi-peak light-curing unit with the light tip in direct contact with the ceramic's outer surface. The specimens from groups EM5-, EM10-, RS5-, and RS10- were stored at 37ºC for 24 hours in distilled water. Groups EM5+, EM10+, RS5+, and RS10+ were exposed to 5,000 thermal cycles (Odeme, Luzern, SC, Brazil) in water baths of 5ºC and 55ºC for 30 seconds each, with a transfer time of 6 seconds between baths. After storage, the PVS molds were sectioned using scalpel blade #15 and removed, and all resin-luting agent cylinders were examined at 40x magnification under a light microscope (Olympus, Tokyo, Japan). Cylinders with irregularities, defects, or failure indications were excluded from the analysis. On a universal testing machine (Instron model 4411, Instron Inc., Canton, MA, USA), a PVC holder containing acrylic resin was positioned. The ceramic specimens were attached to the holder using a cyanoacrylate adhesive (Super Bonder Power Flex, Loctite, Sao Paulo, Brazil), and a 0.2 mm-diameter stainless-steel wire was wrapped around each cylinder of resin luting agent at a given time, aligned with the bonding interface. µSBS test was performed at a speed of 1.0 mm/min until failure. There were no pre-test failures. The de-bonded specimens were examined under a light microscope (Olympus Corp.) and failure modes were classified as adhesive (mode 1), cohesive within the ceramic (mode 2), cohesive within the resin luting agent (mode 3), and mixed (mode 4) involving the resin luting agent and the ceramic.

### Surface morphology by scanning electron microscopy (SEM)

 To analyze the surface morphology of the etched ceramics, one specimen from each HF concentration (5 or 10%) and commercial brand (IPS e.max® CAD and Rosetta® SM) group was finished and polished using silicon carbide sandpapers and immersed in an ultrasonic bath for 10 minutes. The specimens were then mounted on metallic stubs and gold-sputtered (Balz-ers-SCD 050, Balzers Union, Aktiengesellschaft, Furstentun, Liechtenstein) at 40 mA for 180 seconds. The surfaces of the specimens were examined by a single operator using an SEM (LEO 435 VP, Cambridge, UK) at 20 Kv. The specimens were analyzed at magnifications of 1,500x, 5,000x, and 10,000x.

### Statistical analysis

µSBS data were obtained in kgf/cm^2^ and then converted to MPa. For each group, ten ^10^ specimens were tested, and the mean value of the two ^2^ cylinders for each aging (24 h or thermal cycling) of resin luting agent was considered the µSBS value for each specimen. Before the three-way analysis of variance (HF concentration x ceramic x aging), values were examined for normality (Shapiro-Wilk test) and equality of variance (Levene's test), revealing that they were normal. Tukey's post-hoc test (α = 0.05) was used to make multiple comparisons. SPSS (IBM) software was used to conduct the statistical analyses.

## Results

### Microshear bond strength (µSBS)

Table 2 shows the mean µSBS values and standard deviation. The factors evaluated HF concentration (p < 0.0001) and aging (p < 0.0001) influenced µSBS. The factor ceramic (p = 0.085) was not statistically significant. The following interactions between factors were not statistically significant: ceramic / HF concentration (p = 0.358), ceramic / aging (p = 0.136), HF concentration/aging (p = 0.138), and triple interaction (p = 0.610). After 24 hours of storage, the µSBS of the evaluated ceramics was similar (p > 0.05). For both ceramics, etching with 10% HF resulted in higher µSBS (p < 0.05). Regardless of ceramic brand or HF concentration, thermal cycling reduced µSBS when compared to 24 hours (p < 0.05). After thermal cycling at a concentration of 5%, no statistically significant differences were found between ceramics (p > 0.05). IPS e.max® CAD presented higher µSBS than Rosetta® SM when etched by 10% HF (p < 0.05). There were no differences between HF concentrations for Rosetta® SM after thermal cycling (p > 0.05), whereas HF 10% resulted in a higher µSBS for IPS e.max® CAD than HF 5% (p < 0.05). µSBS for IPS e.max® CAD decreased by 33.1% and 32.2% after 24 hours and after thermal cycling, respectively. The reduction for Rosetta® SM was 37.5% and 41.8%, respectively.

### Failure modes

**Figure 2:**
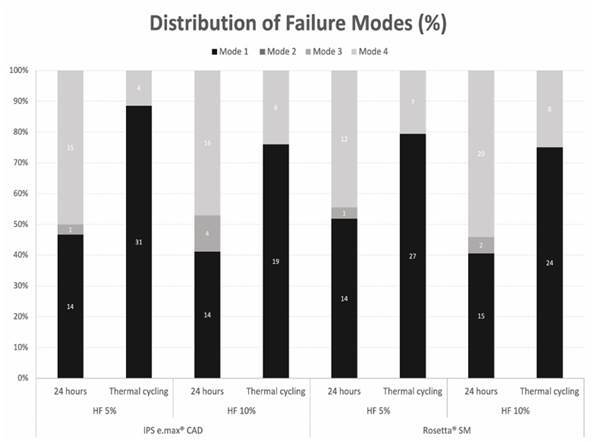
Failure modes of de-bonded specimens (%) according to commercial brand, HF concentration, and aging protocol.

Figure 2 depicts the results for failure modes. After thermal cycling, adhesive failures (mode 1) predominated regardless of HF concentration or ceramic material. Fisher's exact test revealed a statistically significant association with aging (p < 0.0001). At 24 hours, regardless of HF concentration, the majority of failure modes for both ceramics were adhesive (mode 1) and mixed (mode 4). Fisher's exact test did not reveal a significant correlation between failure modes and ceramic type (p = 0.662) or concentration (p = 0.098).

**Table 2:** Mean µSBS (MPa) values and standard deviation (SD) according to aging protocol, ceramic, and hydrofluoric acid (HF) concentration.

		µSBS (MPa)	
Aging	Ceramic	HF 5%	HF 10%
24 hours	IPS e.max® CAD	26.3 (3.8) Ab*	30.4 (3.4) Aa*
	Rosetta® SM	26.4 (3.9) Ab*	29.9 (2.9) Aa*
Thermal cycling	IPS e.max® CAD	17.6 (1.5) Ab	20.4 (2.3) Aa
	Rosetta® SM	16.5 (2.7) Aa	17.4 (2.1) Ba

Means followed by similar uppercase letters are not significantly different within a given column (compares ceramic types). Means followed by similar lowercase letters are not significantly different within a given row (compares HF concentration). Means followed by an asterisk (*) in 24 hours are statistically higher than the corresponding ceramic type within each HF concentration after thermal cycling.

### Surface morphology

**Figure 3:**
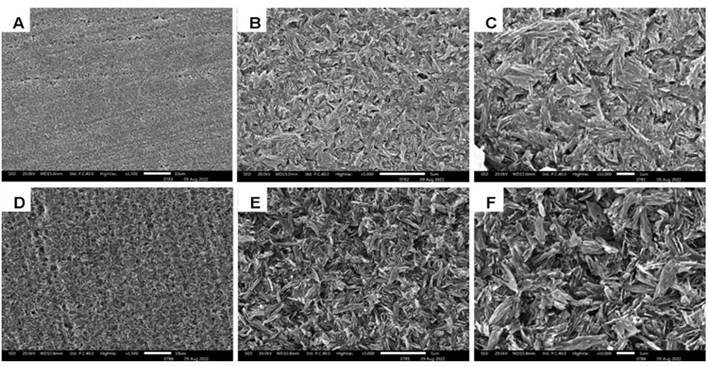
SEM images of IPS e.max® CAD after etching with hydrofluoric acid (HF). A- 5% at 1.500x; B- 5% at 5.000x; C- 5% at 10.000x; D- 10% at 1.500x; E- 10% at 5.000x; F- 10% at 10.000x.

**Figure 4:**
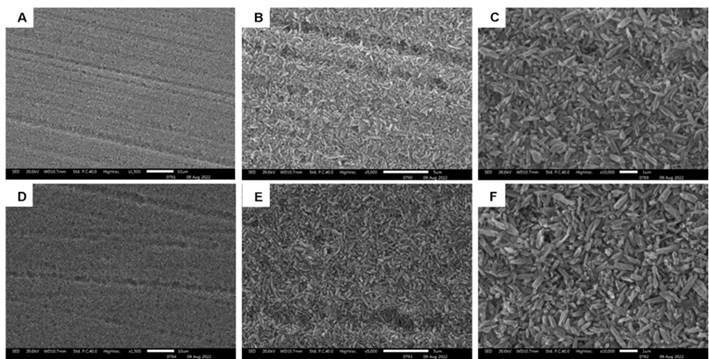
SEM images of Rosetta® SM after etching with hydrofluoric acid (HF). A- 5% at 1.500x; B- 5% at 5.000x; C- 5% at 10.000x; D- 10% at 1.500x; E- 10% at 5.000x; F- 10% at 10.000x.

Figures 3 and 4 show the surfaces of IPS e.max® CAD and Rosetta® SM when etched with 5% or 10% HF, respectively. Concentrations of HF influenced the surface morphology of both ceramics directly. The size and distribution of lithium disilicate crystals in IPS e.max® CAD (Figures 3B, 3C, 3E, and 3F) and Rosetta® SM (Figures 4B, 4C, 4E, and 4F) differ slightly. When using HF at 10% (Figures 3E and 3F), IPS e.max® CAD exhibited etching patterns with greater dissolution of the glassy phase and greater exposure of lithium disilicate crystals (needle-like appearance) than when using HF at 5% (Figures 3B and C). Rosetta® SM revealed a minor difference in the glass phase dissolution and exposure of lithium disilicate crystals between HF 5% (Figures 4B and 4C) and HF 10% (Figures 4E and 4F), with a greater removal of the glassy matrix at 10%.

## Discussion

The first hypothesis of this study, which suggested that various HF concentrations would affect µSBS, was accepted. Our findings indicate that after 24 hours of storage, µSBS for HF 10% was significantly higher than for HF 5%, regardless of the lithium disilicate ceramic brand. After thermal cycling, the µSBS when using HF 10% for IPS e.max® CAD was significantly higher than that of HF 5%, except *between HF 5% and HF 10% for Rosetta® SM after thermal cycling when* µSBS showed no statistical difference. Compared to 10% HF, 5% HF was found to be insufficient for the proper dissolution of the glassy phase in this study, resulting in a decreased surface area and, consequently, surface energy. A smaller contact area between the ceramic surface and the resin luting (lower wetting) agent would reduce their mechanical interlocking and bond strength ^2^ ^,^ ^10^ ^,^ ^11^ ^,^ ^15^. The results of this study do not corroborate the findings of previous research that showed statistically similar results for 5% and 10% HF when applied at room temperature for 20 seconds ^2^ ^,^ ^10^ ^,^ ^11^. This may have occurred because the present study evaluated CAD/CAM ceramics, whereas the cited studies used pressed ceramics. Probably, the difference has been due to slight variations that were observed in the distribution of lithium disilicate crystals between IPS e.max® CAD and Rosetta® SM according to the SEM images (Figures 3 and 4). However, other studies have also shown that the dissolution of the ceramic surface is proportional to the HF concentration used for etching ^2^ ^,^ ^10^ ^,^ ^11^ ^,^ ^24^, which could result in a stronger bond between the ceramic and the resin luting agent, supporting the results of the present study. Studies suggest that the type of ceramic and HF concentration influence bond strength more than the resin luting agent ^2^ ^,^ ^10^ ^,^ ^11^. The HF concentration acts to dissolve the vitreous phase, exposing crystals and resulting in microporosities on the ceramic structure, increased surface area, and improved bonding quality and bond strength ^9^ ^,^ ^14^ ^,^ ^25^. The higher the HF concentration, the greater the dissolution of the glass phase of the ceramic and consequently the bond strength ^2^ ^,^ ^10^ ^,^ ^11^.

The second tested hypothesis, which suggested that the µSBS values for the different ceramics would be comparable, was accepted. At 24 hours or after thermal cycling, there was no significant difference in µSBS between IPS e.max® CAD and Rosetta® SM, except for HF 10% after thermal cycling, when µSBS of IPS e.max® CAD was significantly higher than that of Rosetta® SM. This study found that the bond strength of both lithium disilicate reinforced glass ceramics was comparable to that found in previous studies ^3^ ^,^ ^8^, which did not detect any significant differences. Previous studies have shown that lithium disilicate-based CAD/CAM ceramics (IPS e.max® CAD and Rosetta® SM) exhibit similar characteristics, crystalline pattern, morphological structures, porosity, and mechanical properties ^4^ ^,^ ^8^. Another study has demonstrated that IPS e.max® CAD and Rosetta® SM undergo a close conversion of lithium metasilicates to lithium disilicate crystals during heat treatment as suggested by the manufacturers ^8^. In the present study, however, slight variations were observed in the distribution of lithium disilicate crystals between IPS e.max® CAD (Figures 3B, 3C, 3E, and 3F) and Rosetta® SM (Figures 4B, 4C, 4E, and 4F).

Because aging protocols (thermal cycling) and HF concentration significantly reduced the bond strength of resin luting agent to lithium disilicate ceramics, the third hypothesis, which stated that thermal cycling would affect bond strength, was accepted. Compared to specimens stored for 24 hours, the bond strength of IPS e.max® CAD etched with 5% or 10% HF decreased by 33.1% and 32.1%, respectively. Rosetta® SM's bond strength decreased by 37.5% and 41.8%, respectively. The reduction in bond strength observed in this study after thermal cycling is consistent with the findings of previous studies, which showed that thermal cycling under moist conditions and at varying temperatures significantly reduced the propagation of small cracks at the interface of the ceramic surface and the resin luting agent ^9^ ^,^ ^12^ ^,^ ^26^. Moreover, if materials with varying coefficients of thermal expansion and conductivity are bonded, temperature changes can result in different stresses ^27^. In addition, materials with varying modulus of elasticity can cause stresses at the bonding interface, which eventually contribute to the interface's degradation ^28^.

All of these factors, in conjunction with the effects of hydrolytic degradation caused by thermal cycling, contributed significantly to the observed decrease in bond strength ^29^. The reduction of mechanical properties of resin luting agents as a result of the degradation of the bonding interface due to thermal cycling cannot be ignored as a factor that affects adhesive bond strength ^29^. Due to continuous water sorption, the mechanical properties of resin luting agents decrease ^9^. Previous research has demonstrated that the functional sensitivity of a resin composite is dependent on several factors, including the concentration and type of load, the degree of monomer conversion, and the fraction of intrinsic nanopore volume ^30^ ^,^ ^31^. Moisture, thermal, and mechanical changes, as well as variations in oral pH ^9^ ^,^ ^12^, which can induce physicochemical changes in dental materials ^21^, can influence the durability of the bonding interface between silanized ceramic and resin luting agents. When both ceramics were subjected to a 24-hour bond strength test, regardless of HF concentration, the analysis of failure mode revealed that adhesive failures (mode 1) and mixed failures (mode 4) were predominant. In contrast, more adhesive failures (mode 1) were observed in the groups subjected to thermal cycling, regardless of HF concentration and ceramic type, as a result of inferior adhesive interface quality (Figure 2). Given that HF is toxic and can cause severe lesions in the oral soft tissues, caution should be exercised during clinical practice. Priority should be given to the use of personal protective equipment and well-ventilated rooms to protect health professionals from injury. In addition, future research is required to evaluate aging due to mechanical fatigue, etching times, and various viscosities of the resin-luting agent.

Based on the findings of this study, it is possible to conclude that the bond strength of a resin luting agent to lithium disilicate-reinforced glass ceramics was affected by the HF concentration. IPS e.max® CAD and Rosetta® SM ceramics exhibited similar µSBS values. The bond strength of both ceramics was significantly decreased by thermal cycling.

## References

[1] Moretto G, Pupo YM, Bueno AL, Araujo FO (2016). Prosthetic rehabilitation of a patient with gastroesophageal reflux disease: five-year follow-up. Oper Dent.

[2] Sundfeld D, Naves LZ, Costa AR, Correr AB, Consani S, Borges GA (2015). The effect of hydrofluoric acid concentration on the bond strength and morphology of the surface and interface of glass ceramics to a resin cement. Oper Dent.

[3] Baratto SS, Spina DR, Gonzaga CC, Cunha LF, Furuse AY, Baratto F (2015). Silanated surface treatment: effects on the bond strength to lithium disilicate glass-ceramic. Braz Dent J.

[4] Kang SH, Chang J, Son HH (2013). Flexural strength and microstructure of two lithium disilicate glass ceramics for CAD/CAM restoration in the dental clinic. Restor Dent Endod.

[5] de França DG, Morais MH, das Neves FD, Carreiro AF, Barbosa GA (2017). Precision fit of screw-retained implant-supported fixed dental prostheses fabricated by CAD/CAM, copy-milling, and conventional methods. Int J Oral Maxillofac Implants.

[6] Lim CH, Jang YS, Lee MH, Bae TS (2020). Evaluation of fracture strength for single crowns made of the different types of lithium disilicate glass-ceramics. Odontology.

[7] Sakaguchi RL, Powers JM (2011). Craig's restorative dental materials-e-book. Elsevier Health Sciences.

[8] Tavares LDN, Zancopé K, Silva ACA, Raposo LHA, Soares CJ, Neves FDD (2020). Microstructural and mechanical analysis of two CAD-CAM lithium disilicate glass-reinforced ceramics. Braz Oral Res.

[9] Guarda GB, Correr AB, Gonçalves LS, Costa AR, Borges GA, Sinhoreti MA (2013). Effects of surface treatments, thermocycling, and cyclic loading on the bond strength of a resin cement bonded to a lithium disilicate glass ceramic. Oper Dent.

[10] Puppin-Rontani J, Sundfeld D, Costa AR, Correr AB, Puppin-Rontani RM, Borges GA (2017). Effect of hydrofluoric acid concentration and etching time on bond strength to lithium disilicate glass ceramic. Oper Dent.

[11] Sundfeld D, Correr-Sobrinho L, Pini NIP, Costa AR, Sundfeld RH, Pfeifer CS (2016). Heat treatment-improved bond strength of resin cement to lithium disilicate dental glass-ceramic. Ceramics International.

[12] Salvio LA, Correr-Sobrinho L, Consani S, Sinhoreti MA, de Goes MF, Knowles JC (2007). Effect of water storage and surface treatments on the tensile bond strength of IPS Empress 2 ceramic. J Prosthodont.

[13] Spohr AM, C LC, Consani S, Sinhoreti MA, Knowles JC. (2003). Influence of surface conditions and silane agent on the bond of resin to IPS Empress 2 ceramic. Int J Prosthodont.

[14] Torres SM, Borges GA, Spohr AM, Cury AA, Yadav S, Platt JA (2009). The effect of surface treatments on the micro-shear bond strength of a resin luting agent and four all-ceramic systems. Oper Dent.

[15] Kukiattrakoon B, Thammasitboon K (2007). The effect of different etching times of acidulated phosphate fluoride gel on the shear bond strength of high-leucite ceramics bonded to composite resin. J Prosthet Dent.

[16] Makishi P, André CB, Silva JL, Bacelar-Sá R, Correr-Sobrinho L, Giannini M (2016). Effect of Storage Time on Bond Strength Performance of Multimode Adhesives to Indirect Resin Composite and Lithium Disilicate Glass Ceramic. Oper Dent.

[17] Kalavacharla VK, Lawson NC, Ramp LC, Burgess JO (2015). Influence of etching protocol and silane treatment with a universal adhesive on lithium disilicate bond strength. Oper Dent.

[18] Yoshida F, Tsujimoto A, Ishii R, Nojiri K, Takamizawa T, Miyazaki M (2015). Influence of surface treatment of contaminated lithium disilicate and leucite glass ceramics on surface free energy and bond strength of universal adhesives. Dent Mater J.

[19] Öztürk E, Bolay Ş, Hickel R, Ilie N (2013). Shear bond strength of porcelain laminate veneers to enamel, dentine and enamel-dentine complex bonded with different adhesive luting systems. J Dent.

[20] Lise DP, Perdigão J, Van Ende A, Zidan O, Lopes GC (2015). Microshear bond strength of resin cements to lithium disilicate substrates as a function of surface preparation. Oper Dent.

[21] Oyafuso DK, Ozcan M, Bottino MA, Itinoche MK (2008). Influence of thermal and mechanical cycling on the flexural strength of ceramics with titanium or gold alloy frameworks. Dent Mater.

[22] Aguiar AP, Costa AR, Correr AB, Vedovello SA, Vedovello M, Crepaldi MV (2019). Effect of hydrofluoric acid concentration and thermal cycling on the bond strength of brackets to ceramic. Braz Dent J.

[23] Kim SH, Choi YS, Kang KH, Att W (2022). Effects of thermal and mechanical cycling on the mechanical strength and surface properties of dental CAD-CAM restorative materials. J Prosthet Dent.

[24] Roulet JF, Söderholm KJ, Longmate J (1995). Effects of treatment and storage conditions on ceramic/composite bond strength. J Dent Res.

[25] Hooshmand T, Rostami G, Behroozibakhsh M, Fatemi M, Keshvad A, van Noort R (2012). Interfacial fracture toughness of different resin cements bonded to a lithium disilicate glass ceramic. J Dent.

[26] Borges GA, Caldas D, Taskonak B, Yan J, C LC, de Oliveira WJ (2009). Fracture loads of all-ceramic crowns under wet and dry fatigue conditions. J Prosthodont.

[27] Vásquez V, Ozcan M, Nishioka R, Souza R, Mesquita A, Pavanelli C (2008). Mechanical and thermal cycling effects on the flexural strength of glass ceramics fused to titanium. Dent Mater J.

[28] Yang R, Arola D, Han Z, Zhang X (2014). A comparison of the fracture resistance of three machinable ceramics after thermal and mechanical fatigue. J Prosthet Dent.

[29] Correr-Sobrinho L, Costa AR, Fugolin APP, Sundfeld D, Ferracane JL, Pfeifer CS (2019). Effect of experimental resin cements containing thio-urethane oligomers on the durability of ceramic-composite bonded interfaces. Biomater Investig Dent.

[30] Ferracane JL (1994). Elution of leachable components from composites. J Oral Rehabil.

[31] Soles CL, Yee AF (2000). A discussion of the molecular mechanisms of moisture transport in epoxy resins. Journal of Polymer Science Part B: Polymer Physics.

